# Children and adolescents with psychiatric disorders have high relative leptin levels upon adjustment for sex, BMI, and pubertal status

**DOI:** 10.1007/s00787-025-02921-4

**Published:** 2026-01-05

**Authors:** Nicola Albers, Jochen Antel, Manuel Föcker, Lars Libuda, Judith Bühlmeier, Raphael Hirtz, Jochen Seitz, Anke Hinney, Johannes Hebebrand, Triinu Peters

**Affiliations:** 1 Department of Child and Adolescent Psychiatry, Psychosomatics and Psychotherapy, LVR-University Hospital Essen, University of Duisburg- Essen, Wickenburgstraße 21, Essen, 45147 Germany; 2 Department of Child and Adolescent Psychiatry and Psychotherapy, University of Münster, Münster, Germany; 3 Department for Child and Adolescent Psychiatry, Psychosomatics and Psychotherapy, LWL University Hospital Hamm of the Ruhr University Bochum, Campus Gütersloh, Hamm, Germany; 4 Institute of Nutrition, Consumption and Health, Faculty of Natural Sciences, Paderborn University, Paderborn, Germany; 5 Department of Pediatrics, Division of Rare Diseases and CeSER, St. Josef- Hospital, Ruhr-University Bochum, Bochum, Germany; 6 Division of Pediatric Endocrinology and Diabetology, Department of Pediatrics II, University Hospital Essen, University of Duisburg-Essen, Essen, Germany; 7 Center for Child and Adolescent Medicine, Helios University Hospital Wuppertal, Witten/Herdecke University, Wuppertal, Germany; 8 Section of Molecular Genetics in Mental Disorders, University Hospital Essen, Essen, Germany; 9 Center for Translational Neuro- and Behavioural Sciences, University Hospital Essen, Essen, Germany; 10 Institute of Sex and Gender-Sensitive Medicine, University Hospital Essen, Essen, Germany

**Keywords:** Leptin levels, Psychiatric disorders, Depression, Anxiety, Leptin reference values, Children and adolescents

## Abstract

The relationship between leptin levels and psychiatric disorders has been studied more extensively in adults than in children and adolescents. However, the results are conflicting. We investigated serum leptin levels in children and adolescents (11 to 18.9 years) with psychiatric disorders (*n* = 363). Absolute and relative (body-mass-index (BMI)-, sex- and pubertal-stage-adjusted z-scores using reference values of healthy children and adolescents) leptin levels of different patient groups according to diagnosis were compared. The association between leptin levels and depression (Beck Depression Inventory-II) and anxiety (Child Behavior Checklist and Youth Self Report) was examined using regression analysis. Leptin z-scores were higher in patients with psychiatric disorders than in healthy controls (median 1.50, *p* < .001). While global tests suggested differences in leptin z-scores between patients with different psychiatric disorders, these differences could not be attributed to diagnosis groups in post-hoc pairwise comparisons. Absolute leptin levels differed between psychiatric disorders (*p* < .001). Patients with anorexia nervosa (AN) had the lowest levels, and patients with mood disorders had higher leptin levels than patients with mental disorders other than mood disorders, anxiety or AN. Neither absolute nor relative leptin levels were related to depressive or anxiety symptoms in regression models adjusted for sex and BMI. Significantly elevated BMI-, sex- and puberty-stage-adjusted leptin levels were observed in children and adolescents with psychiatric disorders compared to a reference sample. Further controlled studies are needed to confirm and explain this finding. No relationship was found between absolute or relative leptin levels and symptoms of depression or anxiety.

**Supplementary Information:**

The online version contains supplementary material available at 10.1007/s00787-025-02921-4.

## Introduction

Depression and anxiety disorders are the most common mental disorders in children and adolescents, as well as in adults [1]. Among children and adolescents aged 5–18 in Europe, the prevalence of major depressive disorder (MDD) was 1.7%. The disorder was infrequent in younger children (primary school-aged children: 0.6%) but 4.2 times more common in secondary school-aged children with prevalence of 2.5% [2]. The main criteria for the diagnosis of depression are depressed mood, loss of interest, and inhibition of drive [3]. Secondary criteria include vegetative symptoms such as weight changes, loss of appetite, and sleep disturbances [4]. Among psychiatric disorders in children and adolescents aged 5–18 years in Europe, anxiety disorders had the highest prevalence rate at 7.9% [2].

Biomarkers may be helpful for diagnosing, monitoring disease courses, and assessing treatment response [5]. However, although numerous biomarkers have been linked to psychiatric disorders, they have not yet achieved clinical relevance [6]. Leptin has been proposed as a potential biomarker for depression and stress [7–9]. Leptin plays a central role in regulating food intake by influencing the feeling of satiety [10, 11]. It acts on neurons in the arcuate nucleus of the hypothalamus, where it inhibits orexigenic neuropeptides (NPY, AgRP) and activates anorexigenic POMC neurons, thereby signaling sufficient energy stores and promoting satiety [11]. In conditions such as obesity, however, leptin resistance can impair this signaling, so elevated leptin levels fail to suppress appetite. It is primarily produced in white adipose tissue, and its serum levels correlate with body fat percentage [12]. Because leptin can cross the blood-brain barrier, it can exert central effects [13]. Leptin receptors have been identified in both central and peripheral tissues [14]. In the central nervous system, leptin receptors are located in the hypothalamus, thalamus, and cerebellum, among others [15, 16]. Through the dopaminergic reward system, leptin may influence monoaminergic neurotransmission and, thereby, affect symptoms of depression [14, 17]. More specifically, leptin receptors that are expressed on dopamine neurons in the ventral tegmental area (VTA) modulate dopamine synthesis, release, and receptor activity, all of which are critical for motivation and reward processing – processes that are central to depressive symptomatology [18, 19]. Animal models have shown that leptin administration alleviates depressive-like behaviors through dopamine D1 and D2 receptor pathways, and influences neuroplasticity and neurotrophic factors such as BDNF [20, 21]. Leptin also affects serotonergic and GABAergic signaling, thereby further regulating mood and anxiety symptoms [17, 19]. Clinical data indicate that leptin dysregulation correlates with ‘somatic anxiety’ and depressive symptoms, suggesting leptin’s indirect modulation of key brain circuits involved in emotional processing [20, 22]. While much of this mechanistic understanding originates from animal studies, emerging human data underscore leptin’s potential relevance in mood and anxiety disorders. Through the hypothalamic-pituitary axis, leptin is involved in the regulation of endocrine functioning, including that of glucocorticoids [23]. Studies have found that glucocorticoids increase leptin production, while leptin inhibits glucocorticoid synthesis [24], which may be related to psychiatric disorders. In anxiety and depression, circulating glucocorticoids seem to be elevated [25]. Increased anxiety-related behavior, similar to the behavioral phenotype in depression, has been found in leptin-deficient mice, suggesting a role of leptin in anxiety [14, 26].

Nutritional status affects physiological and behavioral processes in both animals and humans [27]. Accordingly, cognitive, emotional, and behavioral characteristics change during adaptation to starvation, and hypoleptinemia may play an important role in this [14]; Studies have shown that patients with anorexia nervosa (AN) have significantly lower absolute serum leptin levels than people of normal body weight [28]. Clinical observations further suggest that relative leptin deficiency – defined as a lower leptin level than expected for a given weight, sex, and pubertal stage – may also influence mood and weight phobia [29]. Metreleptin use, a recombinant human leptin, in patients with AN [30, 31] has shown potential effects on mood, eating behavior, and cognition [32–34].

The relationship between serum leptin levels and psychiatric disorders has been examined in several clinical studies, but the results are controversial [20]: Studies in adults report inconsistent associations between leptin and depression, with findings ranging from negative to positive correlations, often influenced by BMI adjustment [9, 35–37].

Only a few studies have investigated the relationship between leptin and depression in children and adolescents: Tunçel et al. found no differences in leptin levels unadjusted for BMI between adolescent patients with major depressive disorder and healthy controls [38]. A large cohort study (*n* = 3258) showed that higher leptin levels at the age of 9 predicted later depressive episodes, independent of BMI [39].

Leptin levels have also been studied in relation to anxiety-related disorders. Kim et al. (2019) found no differences in leptin levels between adult patients with MDD or panic disorder and healthy controls, but they did find that higher pre-treatment leptin levels (unadjusted for BMI) predicted improvement in panic symptoms following treatment in patients with depression. However, this effect was not observed in patients with panic disorder [40]. Another study showed that higher levels of phobic anxiety were associated with higher serum leptin levels independent of BMI in women with type 2 diabetes [41]. There are only a few studies on leptin and anxiety in children: Byrne et al. (2019) [42] found no association between leptin levels adjusted for fat mass and anxiety, while Ozmen et al. (2019), using unadjusted leptin levels, also did not find a significant association with anxiety [43].

Because leptin levels depend on BMI, sex, and pubertal stage, relative leptin values (z-scores) compared with a reference population considering these factors can be defined in addition to the absolute leptin values [44]. Such leptin z-scores indicate how many standard deviations the measured serum leptin level deviates from the mean value of a reference population given BMI, sex, and puberty state. To our knowledge, no study has yet examined leptin z-scores in children and adolescents with psychiatric disorders.

We addressed the following research questions: (1) Do serum leptin levels adjusted for BMI, sex, and puberty stage (leptin z-score) in children and adolescents with psychiatric disorders differ from reference values in healthy children? (2) Do serum leptin levels (leptin z-score and absolute leptin level) differ in children and adolescents with different psychiatric diagnoses? (3) Is there an association between leptin levels and symptoms of depression or anxiety, and do the results change if cases with AN are excluded?

The hypothesis is that both elevated and decreased leptin levels (quadratic relationship) can lead to mood disorders. We expect that leptin levels in a group of psychiatric inpatients will differ from those in healthy individuals (question 1). Further, we assume there will be differences between the diagnoses. Specifically, we expect to find reduced leptin levels in patients with AN, even after adjusting for sex, BMI, and Tanner stage, and deviating leptin levels in patients with mood disorders. Based on the relationship described above, we expect a U-shaped relationship between leptin and depression and anxiety, but a linear relationship in both directions is also possible.

## Materials and methods

### Data sources

Children and adolescents aged 11–18.9.9 years with various psychiatric diagnoses who were treated as inpatients or day-care patients at the Department of Child and Adolescent Psychiatry, Psychosomatics and Psychotherapy, LVR-University Hospital Essen, University of Duisburg-Essen, Essen, Germany were included. Data was obtained from two completed studies on nutrition and mental health conducted between 2016 and 2020 [45]. The ‘Vitamin D study’ focused on the effects of vitamin D supplementation using a two-armed parallel-group, double-blind, randomized controlled design. Here, we only used baseline data from this study. The second study was the cross-sectional Nutrition and Mental Health study, which aimed to examine the relationship between diet, nutrient supply, metabolism, and mental health in children and adolescents [46]. Exclusion criteria were a concurrent diagnosis of a severe somatic disease and/or an intelligence quotient below 70. Cases with and without medication were included. At admission or within the preceding 12 months, *n* = 58 patients were treated with antidepressants (*n* = 55 SSRIs, *n* = 3 mirtazapine, *n* = 0 tricyclics), stimulants (*n* = 10), and neuroleptics (*n* = 20). No patient was medicated with barbiturates or phenytoin. Written informed consent was obtained from the children and adolescents and their parents or guardians. Both studies were conducted in accordance with the Declaration of Helsinki and approved by the local ethics committee (No.15–6363-BO).

### Psychological assessment

Psychiatric diagnoses were assessed at admission using the semi-structured interview “Kiddie Schedule for Affective Disorders and Schizophrenia for School Aged Children - Present and Lifetime Version” (K-SADS-PL) according to DSM-IV [47].

The German version of the Beck Depression Inventory-II (BDI-II) was used to assess the severity of depressive symptoms [48, 49].

The anxious/depressed syndrome scales of the Child Behavior Checklist (CBCL) and the Youth Self Report (YSR) were used to measure anxiety [50]. CBCL is a standardized questionnaire for parents. It measures their children’s competencies and problems [50]. YSR is a self-report questionnaire for children and adolescents to assess their competencies and problems in a similar way [50] (see Supplementary Information 1).

Socioeconomic status (SES) was calculated based on occupation, income and parents’ level of education [51, 52].

### Auxiologic parameters

BMI at admission was calculated by dividing the measured body weight by the square of measured height (kg/m^2^) upon admission. Based on nationally representative German reference data for children (KiGGS) [53], individual BMI values were transformed into BMI-Standard Deviation Scores (BMI-SDS) using the method proposed by Cole and Green [54] for smoothing reference centile curves. The method was adapted by Kromeyer-Hausschild et al. [55] to calculate BMI-SDS. The BMI-SDS approximates the deviation of an individual’s BMI from the median of the reference group expressed in units of the standard deviation.

During the physical examination, the pubertal stage was assessed using the Tanner stages for pubescent hair (PH) [56]. Because Tanner stage was missing or its assessment refused in *n* = 52 cases, Tanner stage 5 (adult genitals in size and shape) was assumed for female patients older than 13.4 years and for male patients older than 14.1 years (overall *n* = 38). This assumption was based on the Robert Koch Institute study on sexual maturation in children and adolescents in Germany (*N* = 17,641) [57] showing that the mean age for Tanner stage 5 of PH is 13.4 years for girls and 14.1 years for boys [57]. Patients younger than 13.4 years (girls) or 14.1 years (boys) with missing Tanner stage were excluded from all analyses (*n* = 14).

### Leptin measurement and z-score calculation

Blood samples were collected early in the morning after an overnight fast from an antecubital vein through a short catheter. Whole blood was centrifuged after coagulation, then serum was distributed into aliquots and immediately frozen at −80 °C until analysis. Serum leptin levels were determined by enzyme-linked immunosorbent assay (ELISA) (product E077; charge no. 030220). The Mediagnostic kit E077 measures immunoreactive leptin using two monoclonal antibodies that both bind to leptin and thus generate the measured value (sandwich antibody binding) (Mediagnost GmbH, Reutlingen, Germany). The analytical sensitivity of the Leptin Sensitive ELISA E077 is 0.014 ng/ml, and the assay range is 0.014–50 ng/ml. A comparison of the leptin assays using RIA or ELISA carried out by Mediagnost showed a correlation of *r* =.97 between the two assays and, thus, very high comparability [58]. Measurement of the International Leptin Standard WHO/NIBSC 97/594 by Mediagnost Assays showed that the deviation from the theoretical value of 5 µg/ampoule was less than 10% (Mediagnost, 2019). Serum leptin levels were log-transformed (lg) due to a right-skewed distribution to achieve a normal distribution.

For the comparison of serum leptin levels in hospitalized children and adolescents with healthy children and adolescents, we used reference values analyzed by RIA (radioimmunoassay) [44]. The reference study included 713 healthy participants (312 boys and 401 girls) aged between 5.8 and 19.9 years. None of the individuals exhibited signs of acute or chronic illness, ensuring the reference population represented healthy children and adolescents. Leptin z-scores were calculated according to sex, body mass index (BMI), and Tanner stage using the following formula: Leptin z-score=[ln(leptin)-ln(a)-b*BMI]/d. The constants a, b, and d depend on sex and Tanner stage [44].

### Statistical analysis

For descriptive analyses, mean values and standard deviations were calculated. T-tests were calculated to compare leptin levels between males and females. Correlation analyses between absolute leptin levels (lg-transformed) and leptin z-scores were carried out using Pearson correlation analysis.

As leptin z-scores and leptin (lg) were normally distributed in the total study group, but no longer in separate groups by diagnosis, group comparison analyses were calculated non-parametrically. For the comparison of the z-score against the median value 0 (reference value), the non-parametric test for one sample (Wilcoxon test) was used. The whole sample was tested against 0 and the five diagnostic subgroups (described below) were tested against 0 separately.

As a sensitivity analysis, a univariate general linear model (UNIANOVA) was performed with leptin (z-scores and absolute values in separate analyses) as the dependent variable and psychopharmacological medication (yes/no) as the fixed factor. Estimated marginal means with 95% confidence intervals were obtained, and pairwise comparisons were used to assess group differences. This analysis was performed for all patients (*n* = 338) upon exclusion patients with restrictive AN, i.e. based on patients who had not taken any psychopharmacological medication (*n* = 265) and patients who had taken psychopharmacological medication in the last 12 months (*n* = 73, ≥ 1antidepressant (*n* = 56), stimulant (*n* = 10), and/or antipsychotic (*n* = 19)). The groups were also compared in leptin levels (ln). Patients with restrictive AN were excluded from sensitivity analyses due to the hypoleptinemia resulting from starvation, which can confound the assessment of psychotropic medication effects on leptin levels. Additionally, severe underweight and altered metabolic state in AN may impact the efficacy and pharmacodynamics of psychotropic drugs, further complicating interpretation [14, 59–63].

Subgroup analyses of leptin z-scores and absolute leptin levels (lg) were performed with five subgroups. The groups were divided into cases with mood disorders, cases with anxiety disorders, cases with mood disorders and anxiety disorders, cases with AN, and cases with psychiatric disorders other than mood disorders, anxiety disorders, and AN. The mood disorders subgroup includes cases that had mild, moderate, or severe major depression without psychotic symptoms according to DSM-IV (code 296.21, 296.22, 296.23). The anxiety disorders subgroup includes cases with unspecified anxiety disorder (code 300.00), panic disorder with or without agoraphobia (code 300.01, 300.21), generalized anxiety disorder (code 300.02), agoraphobia (code 300.22), social phobia (code 300.23), specific phobia (code 300.29) or separation anxiety disorder (code 309.21) according to DSM-IV.

The subgroup AN includes children and adolescents with AN, i.e., an AN diagnosis according to DSM-IV (code 307.1) and a BMI percentile below 10. Patients with a diagnosis of anxiety disorders or mood disorders in addition to AN were excluded from the subgroup analysis. Non-parametric Kruskal-Wallis one-way analysis of variance (ANOVA) was used for statistical analysis of general differences in leptin z-scores and absolute leptin levels. Regression analyses were calculated to examine the association between leptin levels and severity of depressive and/or anxious symptoms (*N* = 363). The dependent variables were BDI-II, “anxious/depressed” scale of CBCL, or “anxious/depressed” scale of YSR, and predictor variables were age, sex, BMI and absolute leptin level (lg-transformed) (see models 1–3 in the supplementary information 2). SES was tested as additional predictor in these models, but finally not included as it was not significantly associated with the outcomes.

In the sensitivity analysis, the leptin z-score was used as a predictor instead of leptin (lg) (see models 8–10 in the supplementary information 2).

To test whether the effect of leptin levels on depressive and/or anxious symptoms depends on sex or BMI, we further included interaction terms of BMI x leptin level (lg) and sex x leptin level (lg) (see models 4–6 in the supplementary information 2).

In the further sensitivity analysis, the six models were additionally performed without patients with AN (*N* = 338).

To test for a quadratic relationship, the model with BDI-II as dependent variable and age, sex, BMI, leptin level (lg), and the quadratic term of leptin level (lg) as independent variables was calculated (see model 7 in the supplementary information 2).

Exact two-sided significances were calculated. Instead of correcting for multiple testing, we set the alpha level at 0.005. Results with 0.005 < *p* <.05 were reported as suggestive evidence according to Benjamin and colleagues (2018) who proposed these p-value thresholds since a two-sided p-value of 0.005 corresponds to Bayes factors of approximately 14 to 26 in favor of the alternative hypothesis (H_1_) [64]. The above range, thus, represents ‘substantial’ to ‘strong’ evidence according to conventional Bayes factor classifications. The Bonferroni correction was only applied to post-hoc tests in the ANOVA (10 tests).

IBM SPSS Statistics v.27 was used for statistical analysis. PROCESS macro by Andrew F. Hayes was used for the moderation analyses [65].

## Results

### Descriptive statistics

A total of 363 children and adolescents (69.4% female) were included. Descriptive data are presented in Table 1. The mean age was 15.7 years. Mean absolute leptin levels (lg) were higher in girls than in boys (t(193.02)=−7.58, *p* <.001) (Supplementary Fig. 1). Absolute leptin level (lg) and leptin z-score were weakly correlated (*r* =.19; *p* <.001).

**Table 1:** Descriptive statistics (total number of cases; *n* = 363)

Age [vears]*	15.7 (1.58) [11.3–18.9]
Female: N (%)	252 (69.4)
BMI [kg/m2]*	22.9 (6.14) [13.8–54.7]
BMI-SDS *	0.02 (1.53) [−4.9−3.1]
Less than − 2 SD	37 (10.2%)
Between − 2 and − 1 SD	45 (12.4%)
Between − 1 and + 1 SD	175 (48.2%)
Between + 1 and + 2 SD	72 (19.8%)
More than + 2 SD	34 (9.4%)
Tanner stage *	4.3 (0.98) [1–6]
Serum leptin level [ng/ml]*	19.7 (22.86) [0.2–136.8]
Leptin z-score*	1.2 (1.96) [−8.7−6.1]

* values are mean +- SD and ranges

### Comparison of relative leptin levels (z-scores) and absolute leptin levels

#### Relative leptin levels

The Wilcoxon test showed that children and adolescents with psychiatric disorders had a significantly higher leptin z-score (*N* = 363; median 1.50 *p* <.001) than the reference population. Sensitivity analyses showed that there was no effect of psychopharmacological medication on leptin levels (z-score) (Supplementary Information 3).

We compared the leptin z-score of girls and boys against the reference value 0. Patients with AN were excluded from this analysis (*n* = 338). Boys had a higher median leptin z-score (median = 1.81) than girls (median = 1.40). Both values were significantly different from 0 (*p* <.001) (Supplementary Fig. 1).

**Fig. 1:**
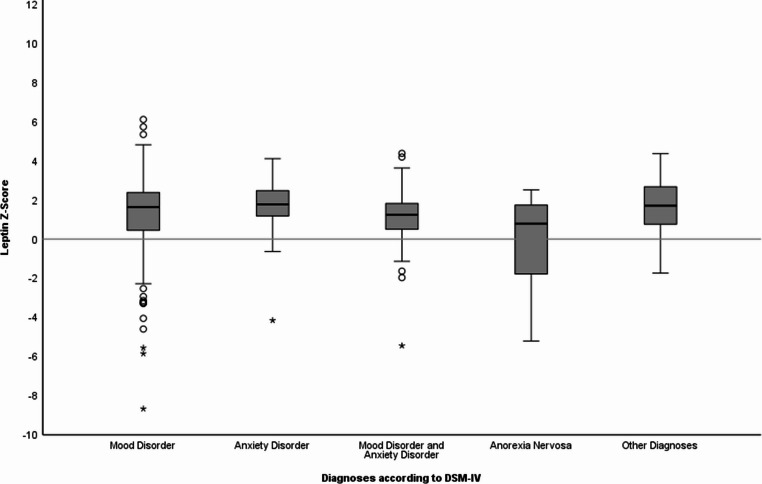
Boxplot of leptin level adjusted for BMI, sex, and puberty stage (z-scores) according to the reference group, divided into five groups according to DSM-IV diagnoses The mood disorders subgroup (*n* = 192) showed median leptin z-score 1.64, the difference from 0 *p* <.001; the anxiety disorder subgroup (*n* = 35) had median leptin z-score 1.77, the difference from 0 *p* <.001; the subgroup mood disorders and anxiety disorders (*n* = 57) had median leptin z-score 1.25, the difference from 0 *p* <.001; the subgroup anorexia nervosa (AN) (*n* = 15) had median leptin z-score 0.79, difference from 0 *p* =.955; the subgroup other diagnoses (*n* = 54) showed median leptin z-score 1.71, *p* <.001. There was a suggestive difference between the groups in leptin z-scores (*p* =.027)

**Fig. 2:**
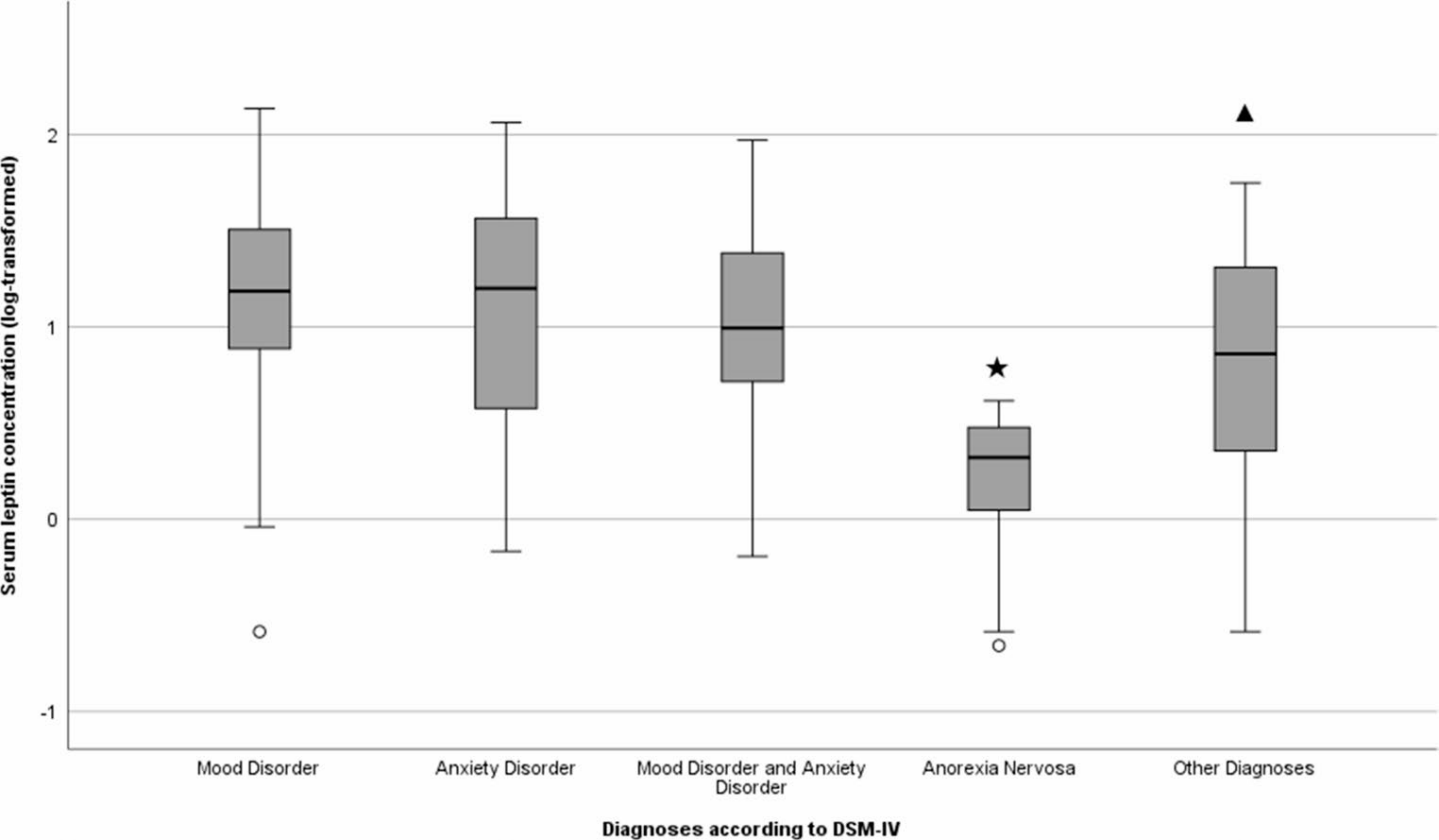
Boxplot of serum leptin level (lg) divided into five groups according to DSM-IV diagnoses: mood disorders (*n* = 192), anxiety disorders (*n* = 35), mood disorders and anxiety disorders (*n* = 57), anorexia nervosa (AN) (*n* = 15) and other diagnoses (*n* = 54) Big black star: patients with AN had lower absolute leptin levels (lg) than all other patients (highest Bonferroni corrected *p* =.013 with the group “other diagnoses”, lowest p-value *p* <.001 in comparison with patients with mood disorders; Big blue triangle: patients “other diagnoses” had lower absolute leptin levels than patients with mood disorders (Bonferroni corrected *p* =.002)

Each diagnosis group (a) cases with mood disorders (*n* = 192), b) cases with anxiety disorders (*n* = 35), c) cases with AN (*n* = 15), d) cases with mood disorders and anxiety disorders (*n* = 57), and e) cases with other psychiatric disorders (without mood disorders, anxiety disorders, and AN) (*n* = 54)) was tested against 0. All diagnostic groups, except the group of patients with AN, had leptin levels significantly higher than expected for given BMI, sex, and pubertal stage (leptin z-scores were significantly different from 0). Leptin z-scores of patients with AN did not differ from 0 (Figs. 1 and 2; Table 2).

**Table 2:** Serum leptin levels ng/ml and leptin level adjusted for BMI, sex, and puberty stage (z-scores) stratified by diagnosis (cases with anorexia nervosa (AN) with comorbidities for mood disorders and/or anxiety disorders are excluded; *n* = 353)

Diagnosis according to DSM-IV	Serum leptin level (ng/ml) mean (SD) [range]	Leptin z-score mean (SD) [range]
Mood Disorder (*N* = 192)	23.2 (24.24) [0.3–136.8]	1.3 (1.97) [−8.7−6.1]
Anxiety Disorder (*N* = 35)	24.6 (28.04) [0.7–115.6]	1.6 (1.48) [−4.2−4.1]
Mood Disorder and Anxiety Disorder (*N* = 57)	19.3 (21.87) [0.6–93.6]	1.1 (1.63) [−5.5−4.4]
Anorexia nervosa (*N* = 15)	2.1 (1.21) [0.2–4.1]	−0.2 (2.39) [−5.2−2.5]
Other Diagnoses (*N* = 54)	12.6 (13.75) [0.3–56.1]	1.6 (1.28) [−1.7−4.4]

Subgroup analysis comparing the five diagnosis groups with each other showed a suggestive significant difference between the groups in leptin z-scores (H(4) = 10.98; *p* =.027). In the post-hoc analyses, it was not possible to show between which groups the differences existed due to the adjustment for multiple testing (Fig. 1).

#### Absolute leptin levels

The differences between the subgroups in absolute leptin levels (lg) were significant (H(4) = 42.44; *p* <.001) (Table 2; Fig. 1). Post-hoc analyses showed that the patients with AN had significantly lower absolute leptin levels (lg) than all other patients. It was also found that patients with mood disorders had higher absolute leptin levels (lg) than patients in the group “other diagnosis”.

### Relationship between leptin and depression/anxiety

Different regression models were used to explain the variance of BDI-II and anxious/depressed scores of CBCL/YSR. Model (1) explained 15.3% of the variance in depression (BDI-II) (Table 3a), which is considered a moderate goodness of fit. Leptin level (lg) did not show a significant association with BDI-II score.

**Table 3:** Results of regression analysis with total BDI-II and the scale “anxious/depressed” of CBCL (*n* = 363) or YSR (*n* = 363) as dependent variable

**a) Results of regression analysis with total BDI-II as dependent variable (model 1)**					
Predictor	B [95%CI]	SE	Beta	T	Sig. ^a^
Step 1 (*R*^*2*^ = 0.146)					
Constant	−1.087 [−13.928−11.754]	6.530		−0.166	0.868
Sex	9.196 [6.568–11.824]	1.336	0.336	6.881	** < 0.001**
Age	1.256 [0.489–2.023]	0.390	0.157	3.221	**0.001**
BMI	0.077 [−0.120−0.274]	0.100	0.037	0.768	0.443
Step 2 (*R*^*2*^ = 0.153)	(F(4, 358) = 16.21, *p* <.001)				
Constant	0.111 [−12.765−12.987]	6.547		0.017	0.987
Sex	7.618 [4.449–10.787]	1.611	0.279	4.728	**< 0.001**
Age	1.360 [0.587–2.134]	0.393	0.171	3.458	**0.001**
BMI	−0.145 [−0.463−0.174]	0.162	−0.070	−0.894	0.372
Leptin level (lg)	3.324 [−0.429−7.077]	1.908	0.149	1.742	0.082
**b) Results of regression analysis with the t-score of the “anxious/depressed” scale of the CBCL as dependent variable (model 2).**					
Predictor	B [95%CI]	SE	Beta	T	Sig. ^a^
Step 1 (*R*^*2*^ = 0.036)					
Constant	60.292 [49.967–70.616]	5.250		11.484	**< 0.001**
Sex	2.955 [0.842–5.068]	1.075	0.143	2.750	0.006
Age	0.336 [−0.281−0.953]	0.313	0.056	1.072	0.285
BMI	0.163 [0.004–0.332]	0.081	0.105	2.019	0.044
Step 2 (*R*^*2*^ = 0.046)	F(4, 358) = 4.27, *p* =.002				
Constant	61.346 [51.003–71.690]	5.260		11.663	**< 0.001**
Sex	1.566 [−0.979−4.112]	1.294	0076	1.210	0.227
Age	0.428 [−0.194−1.050]	0.316	0.071	1.354	0.176
BMI	−0.032 [−0.288−0.223]	0.130	−0.249	−0.249	0.803
Leptin level (lg)	2.927 [−0.088−5.941]	1.533	0.173	1.909	0.057
**c) Results of the regression analysis with the t-score of the “anxious/depressed” scale of the YSR as dependent variable (model 3).**					
Predictor	B [95%CI]	SE	Beta	T	Sig. ^a^
Step 1 (*R*^*2*^ = 0.078)					
Constant	48.202 [36.203–60.200]	6.101		7.901	**< 0.001**
Sex	5.480 [3.024–7.936]	1.249	0.223	4.388	**< 0.001**
Age	1.028 [0.312–1.745]	0.364	0.143	2.822	0.005
BMI	0.113 [−0.072−0.297]	0.094	0.061	1.202	0.230
Step 2 (*R*^*2*^ = 0.091)	F(4, 358) = 8.94, *p* <.001				
Constant	49.620 [37.620–61.621]	6.102		8.132	**< 0.001**
Sex	3.611 [0.658–6.564]	1.502	0.147	2.405	0.017
Age	1.152 [0.431–1.873]	0.367	0.161	3.142	**0.002**
BMI	−0.150 [−0.447−0.147]	0.151	−0.081	−0.994	0.321
Leptin level (lg)	3.937 [0.440–7.434]	1.778	0.196	2.214	0.027
**d) Results of regression analysis with moderation analysis with total BDI-II as dependent variable (model 4).**					
Predictor	B [95%CI]	SE		t	Sig. ^a^
Constant	9.786 [−4.055−23.626]	7.038		1.391	0.165
Leptin level (lg)	−0.120 [−5.200−4.961]	2.583		−0.046	0.963
BMI	0.270 [−0.220−0.759]	0.249		1.084	0.279
Leptin level (lg) x BMI	−0.567 [−1.017- −0.116]	0.229		−2.472	0.014
Sex	9.133 [5.690–12.577]	1.751		5.216	**< 0.001**
Leptin level (lg) x sex	1.320 [−3.380−6.019]	2.390		0.552	0.581
Age	1.164 [0.315–2.013]	0.432		2.697	0.007
*R*^*2*^ = 0.170					
**e) Results of regression analysis with moderation analysis with the t-score of the “anxious/depressed” scale of the CBCL as dependent variable (model 5).**					
Predictor	B [95%CI]	SE		t	Sig. ^a^
Constant	65.651 [55.198–76.105]	5.315		12.351	**< 0.001**
Leptin level (lg)	2.009 [−1.930−5.049]	2.003		1.003	0.316
BMI	0.076 [−0.305−0.460]	0.193		0.391	0.696
Leptin level (lg) x BMI	−0.151 [−0.522−0.221]	0.189		−0.797	0.421
Sex	1.862 [−0.873−4.597]	1.391		1.339	0.182
Leptin level (lg) x sex	−0.229 [−4.228−3.770]	2.033		−0.112	0.910
Age	0.388 [−0.257−1.034]	0.328		1.183	0.237
*R*^*2*^ = 0.048					
**f) Results of regression analysis with moderation analysis with the t-score of the “anxious/depressed” scale of the YSR as dependent variable (model 6).**					
Predictor	B [95%CI]	SE		t	Sig. ^a^
Constant	56.083 [44.049–68.118]	6.119		9.165	**< 0.001**
Leptin level (lg)	1.387 [−2.646−5.419]	2.050		0.676	0.499
BMI	0.161 [−0.251−0.572]	0.209		0.767	0.443
Leptin level (lg) x BMI	−0.420 [−0.810−0.030]	0.198		−2.116	0.035
Sex	4.897 [1.888–7.905]	1.530		3.201	**0.001**
Leptin level (lg) x sex	1.854 [−2.531−6.239]	2.230		0.832	0.406
Age	0.988 [0.242–1.734]	0.379		2.605	0.010
*R*^*2*^ = 0.103					
**g) Results of regression analysis with total BDI-II as dependent variable (model 7).**					
Predictor	B [95%CI]	SE	Beta	T	Sig. ^a^
Step 1(*R*^*2*^ = 0.146)					
Constant	−1.087 [−13.928−11.754]	6.530		−0.166	0.868
Sex	9.196 [6.568–11.824]	1.336	0.336	6.881	**< 0.001**
Age	1.256 [0.489–2.023]	0.390	0.157	3.221	**0.001**
BMI	0.077 [−0.120−0.274]	0.100	0.037	0.768	0.443
Step 2 (*R*^*2*^ = 0.154)	F(5,357) = 12.96 *p* <.001				
Constant	−0.234 [−13.323−12.856]	6.656		−0.035	0.972
Sex	7.675 [4.480–10.869]	1.624	0.281	4.725	**< 0.001**
Age	1.349 [0.571–2.127]	0.396	0.169	3.411	**0.001**
BMI	−0.121 [−0.476−0.233]	0.180	−0.059	−0.672	0.502
Leptin level (lg)	3.362 [−0.404−7.128]	1.915	0.151	1.756	0.080
Leptin level (lg)^2^	0.000 [−0.001−0.001]	0.000	−0.020	−0.299	0.765

*B* unstandardized regression coefficient; *CI* confidence interval; *SE* standard error from unstandardized regression coefficient; *Beta* standardized coefficient (in moderation analysis the standardized beta can not be reported); *t* test statistic; *Sig*. significance; *R*^*2*^ coefficient of determination; *F *F-test of overall significance ^a^ p-values < 0.005 are marked bold

Replacing leptin (lg) with leptin z-score, the results did not change substantially (Table S1). This association was also not significant after excluding patients with AN (leptin (lg): *p* =.539, data not shown).

Model (2) was significant in explaining the variance of the CBCL subscale, but the goodness of fit was low (Table 3b). Leptin level (lg) was not a significant predictor (Table 3b), which was also seen after excluding patients with AN (*p* =.262, further data not shown). When leptin (lg) was replaced by leptin z-score as the independent variable, the results remained almost the same (Table S1). The model (3) with the t-value of the YSR anxiety/depression scale as the dependent variable showed a low goodness of fit (Table 3c). Leptin level (lg) as predictor was suggestively significant (*p* =.027), but not after excluding patients with AN (*p* =.364, more data not shown). Leptin z-score as predictor in model (3) was also not significant (Table S1).

The interaction between leptin level (lg) and sex and between leptin level (lg) and BMI were also tested. The interaction terms between sex and leptin level (lg) were not significant in any model, even after excluding patients with AN (Table 3d-f). The interaction term between BMI and leptin level (lg) revealed suggestive significance in BDI-II (*p* =.014) and YSR anxiety/depression scores (*p* =.035).

The regression model (7) showed no significant quadratic relationship between leptin level (lg) and BDI-II (*p* =.765) (Table 3g).

## Discussion

This study investigated the relationship between leptin levels and the severity of depression or anxiety in a large sample of 363 children and adolescents with psychiatric disorders. To the best of our knowledge, no comparable study of this size has been performed. Moreover, this is the first study to use relative leptin z-scores, which are the deviation from the expected leptin level for a given sex, BMI and pubertal stage according to a reference population. The main results of our study are the following:

(1) We found significantly increased leptin z-scores, except of patients with AN compared with the reference population. (2) This finding was more pronounced in boys than girls. (3) It was not possible to delineate patient groups with apparently higher or lower leptin z-scores. (4) We did not find a relation between absolute or relative leptin levels and symptoms of anxiety or depression.

### Higher relative (z-score) and absolute leptin levels in psychiatric disorders

The magnitude of the higher leptin level than predicted based on sex, BMI, and pubertal stage in children and adolescents with psychiatric disorders compared to the healthy reference population was surprising. BMI has a significant effect on serum leptin levels, with fat mass being the most important determinant here [44]. As expected, girls had higher mean absolute leptin levels than boys. This can be explained by the higher percentage of body fat and by the higher estrogen and lower testosterone levels compared with males [44, 66, 67], but also by the pattern of body fat distribution. Subcutaneous fat expresses more leptin mRNA than visceral fat, particularly in women [68]. Therefore, if our sample of children and adolescents with psychiatric disorders had a higher percentage of fat or differences in body fat distribution than the BMI-matched reference population – potentially resulting from lower physical activity levels [69] – this could account for the elevated leptin z-score relative to the reference population. Data on body composition and physical activity were not available in the present study, and to our knowledge, no studies have specifically examined body composition in children and adolescents with psychiatric disorders. Thus, further studies on leptin in children and adolescents should also take into account body composition rather than just BMI, in addition to sex and pubertal status. The elevated leptin z-scores in a psychiatric sample may also be driven by other factors. For example, antipsychotic drugs such as olanzapine can increase leptin levels [70–73], but our analysis did not show an effect of psychopharmacological treatment on leptin levels.

Absolute serum leptin levels showed expected group differences. Patients with anorexia nervosa (AN) had lower absolute leptin levels than patients with mood and/or anxiety disorders as well as patients without mood or anxiety disorders [28]. In contrast, patients with mood disorders exhibited higher absolute leptin levels than patients with other psychiatric disorders.

Sample preparation or analytical methods are unlikely to have affected these results. Serum leptin levels in our study were determined by ELISA (E077). It is recommended to determine serum leptin levels by either radioimmunoassay (RIA) or ELISA in both children and adults [74]. Blum et al. employed RIA to establish reference values [44], and Carlson et al. (1999) demonstrated that leptin concentrations obtained by RIA and ELISA were essentially indistinguishable [75]. Comparative analyses by Mediagnost GmbH demonstrated a strong correlation between RIA or ELISA results (*r* =.97) with deviations of less than 10% from the theoretical value, indicating a very high degree of comparability [58].

Because only healthy people were included in the Blum et al. study to establish reference values, extrapolation was necessary for very high and very low BMI values. Therefore, leptin z-scores for extreme BMI values should be interpreted cautiously [44]. In our sample, there were *n* = 43 patients with a BMI >30 whose median leptin z-score was 0.19 (this value is based on extrapolated reference data from Blum et al.). The sample without extreme BMI values (*n* = 295), excluding patients with a BMI >30 (*n* = 43) and a diagnosis of AN (*n* = 25), had a median leptin z-score of 1.69. This means that the extreme BMI values in our patient group lowered the overall median for leptin z-scores rather than increasing it and therefore do not explain the higher median value observed in the overall group.

It remains to be discussed whether leptin resistance could explain the observed elevated leptin z-scores in children and adolescents with psychiatric disorders in our study. Progressive loss of fat tissue during starvation could lead to psychological and behavioral effects through absolute hypoleptinemia [14], and recombinant leptin substitution appears to have positive effects on mood and behavior not only in AN but also in patients with atypical AN without absolute leptin deficiency [29, 32, 33]. Whether relative hypoleptinemia, i.e., leptin levels lower than expected for weight, sex, and pubertal stage, also has an effect is the subject of discussion [29]. Obese individuals often have elevated serum leptin levels and a low response to leptin [76]. Our findings raise the possibility that leptin resistance may also occur in psychiatric populations independent of adiposity, potentially leading to elevated circulating leptin levels yet diminished leptin signaling in target neurons. This hypothesis underscores a potentially important pathophysiological mechanism in psychiatric disorders that warrants further investigation, particularly regarding its clinical implications. Overall, leptin resistance is relatively little studied and is still far from being understood, even in human obesity [77]. It may arise from several mechanisms, including abnormalities in one or more of the six known leptin receptor isoforms, impaired intracellular signaling, or disturbances occurring downstream of leptin target neurons. It has also been suggested that leptin resistance could relate to impaired transport of leptin across the blood-brain barrier or defects in downstream neural circuits. Taken together, the current evidence suggests that the multifactorial etiology of leptin resistance involves both receptor-level factors and alterations further along the leptin signaling cascade [78, 79]. In plasma, only the soluble isoform of the leptin receptor (Ob-Re) is measurable, but not the other leptin receptor isoforms (membrane-bound Ob-Ra to Ob-Rf) [80]. The ratio of serum leptin level and soluble receptor (Ob-Re), the free leptin index, has been used to quantify leptin residence in obese individuals [81]. This would be a worthwhile subject for future studies. The hypothesis of a particular type of leptin resistance in some psychiatric disorders could be supported by the finding that the Suppressor of Cytokine Signaling Three (SOCS3) appears to play a role in leptin signaling [77] by inhibiting the JAK2/STAT3 pathway [82]. For example, high leptin levels could attenuate leptin signaling through SOCS3 expression [83]. In one study, SOCS genes were upregulated in people with bipolar disorder compared to healthy individuals [84]. In another study on patients with MDD, a decreased expression of SOCS3 was observed [85]. It could be hypothesized that possible leptin resistance in psychiatric disorders is mediated by SOCS. To test the hypothesis of leptin resistance in mood disorders, studies should include both patients with mood disorders and healthy control groups, and, where feasible, assess leptin receptor expression. Further research into the underlying mechanisms of leptin resistance could provide valuable insights for identifying potential biomarkers and, ultimately, for developing novel treatment approaches.

### Sex differences in absolute and relative leptin levels in psychiatric disorders

Notably, the mean deviation of the leptin levels in boys from the reference value (z-score) was higher than that in girls, even after excluding cases with AN. However, the absolute leptin level was lower in boys. It can be concluded that the leptin levels of boys undergoing psychiatric treatment deviate significantly more from the expected value than those of girls.

In addition, our finding that absolute leptin levels were only weakly correlated with relative leptin levels (z-score) should be taken into account in research on leptin, as absolute and relative measurements appear to be partially independent and are likely to reflect different physiological causes and effects. Absolute leptin levels primarily reflect fat mass, correlating strongly with body fat percentage and BMI [44, 86], yet substantial inter-individual variability exists at comparable adiposity levels [87]. In contrast, relative leptin values provide insight into hormonal dysregulation, leptin resistance, or pathological states that are not apparent from absolute levels alone.

### Depression symptoms and leptin

While we found that patients with mood disorders had higher absolute leptin levels (lg) than patients without mood disorders, we found no association between absolute or relative leptin levels and depression severity after adjusting for weight (BMI), sex, and age in regression models.

Weight is associated with depression — indeed, weight changes are a criterion for diagnosing depression [88] — and weight is an important determinant of serum leptin levels. Nevertheless, in our study, the effects of serum leptin level and BMI on BDI-II score were not significant in models with age and sex as further independent variables. In models with BDI-II and an “anxious/depressed” score of YSR as an outcome, the interaction term of leptin (lg) with BMI revealed suggestive significance only. The quadratic effect of leptin was not significant. This is consistent with the findings of Donnelly et al. that the leptin x BMI interaction terms, quadratic terms for leptin and BMI, nonlinear splines to leptin, BMI, or their interaction had no effect on depression. The model with additive linear terms for leptin and BMI had the best fit [39].

### Anxiety symptoms and leptin

The anxious/depressed subscale of the CBCL and YSR was used to assess anxiety. Although these questionnaires assess various competencies in children and adolescents, they are not specific to the trait of anxiety. A relationship between the anxiety/depression subscale and leptin levels was not found in this study. Controversial results on leptin and anxiety can be found in the literature [41, 89]. Few studies have investigated the relationship between anxiety and leptin in children and adolescents. No association was found between the character trait anxiety, anxiety symptoms or anxiety disorders, and serum leptin levels [42, 43, 90].

### Limitations

Comorbidities of children and adolescents in inpatient psychiatric treatment make it difficult to distinguish between anxiety and depression. Furthermore, independent effects of atypical depression could not be investigated in our study because this subtype of depression was not recorded.

A potential limitation is the use of reference data from 1997 [44]. Since the 1990 s, obesity rates have risen worldwide; in Germany, though, this upward trend appears to have stabilized over the last two decades [91]. Reported shifts in pubertal timing are modest, with international reviews indicating a slightly earlier onset in girls [92]. In contrast, German data suggest stability: the mean age at menarche has remained approximately 12.8 years over recent decades and no relevant changes have been observed in boys [93]. It should also be noted that the equations derived from the reference sample already adjusted for BMI as well as puberty stage. Accordingly, secular changes in BMI are per se should not affect leptin z-score calculation. Given that leptin concentrations in our study deviated by more than one standard deviation from expected values, minor secular changes in reference data are unlikely to explain these findings.

When calculating leptin z-scores, some extreme BMI values in our study group should be noted. The reference values provided by Blum et al. only refer to extrapolated values for the marginal values. However, these do not appear to be the source of the high mean value in our study group. In addition, the pubertal development of pubic hair was included in the calculation of the z-scores, but this is only an indirect marker of pubertal stage.

Our study used a problem scale from the CBCL and YSR to investigate trait anxiety. However, for a more meaningful assessment of anxiety, a stand-alone questionnaire such as the State-Trait Anxiety Inventory for Children would be more meaningful.

We performed cross-sectional measurements, so we could not investigate causal relationships.

## Conclusions

In this study, a significant increase in relative serum leptin levels upon adjustment for sex, BMI and pubertal status was observed in children and adolescents with psychiatric disorders compared with a reference sample, which was more pronounced in boys. Further controlled studies are needed to explain this increase. Relative leptin levels should be taken into account in future studies of leptin in children and adolescents. A relationship between leptin and depression symptoms or leptin and anxiety symptoms could not be found in the present sample.

Group differences in absolute leptin levels, such as elevated levels in mood disorders and reduced levels in anorexia nervosa, likely reflect underlying categorical disease states diagnosed clinically, whereas trait-based analyses capture symptom severity on a continuous scale that is apparently not directly aligned with leptin concentrations.

Long-term prospective studies would be useful to clarify the causal relationship between absolute and relative leptin levels with psychiatric disorders.

## Supplementary Information

Below is the link to the electronic supplementary material.

Supplementary File 1 (DOCX 71.9 KB)

## Data Availability

No datasets were generated or analysed during the current study.
