# Supplementary material for: Children and adolescents with psychiatric disorders have high relative leptin levels upon adjustment for sex, BMI, and pubertal status
Source: Eur Child Adolesc Psychiatry. 2026 Jan 5;35(5):1451–65. doi: 10.1007/s00787-025-02921-4 (PMC13272241; doi:10.1007/s00787-025-02921-4)
Supplement: Supplementary file 1 — (DOCX 71.9 KB) [file 787_2025_2921_MOESM1_ESM.docx]

**Children and adolescents with psychiatric disorders have high relative leptin levels upon adjustment for sex, BMI, and pubertal status**

Supplementary Information 1.

**Psychological assessment**

Psychiatric diagnoses were assessed at admission using the semi-structured interview “Kiddie Schedule for Affective Disorders and Schizophrenia for School Aged Children - Present and Lifetime Version” (K-SADS-PL) according to DSM-IV [47].

The German version of the Beck Depression Inventory-II (BDI-II) was used to assess the severity of depressive symptoms. The BDI-II is a 21-item self-report questionnaire, each rated on a four-point Likert scale (0–3 points). The lowest possible score is 0, and the highest is 63 [48,49]. The higher the score, the more severe the depression. From 9 to 13 points, the depression is classified as minimal, up to 19 points as mild, up to 28 points as moderate, and up to 63 points as severe [49].

The anxious/depressed syndrome scales of the Child Behavior Checklist (CBCL) and the Youth Self Report (YSR) were used to measure anxiety [50]. The CBCL is a standardized questionnaire for parents. It measures their children's competencies and problems [50]. The 120 items are scored from 0 to 2. They are divided into six syndrome scales. Scale scores were standardized for sex and age according to the German reference group (T-values) [51]. In addition, the items can be divided into externalizing and internalizing, as well as into competencies "activity”, “social relations” and “school“. The YSR is a self-report questionnaire for children and adolescents to assess their competencies and problems in a similar way [50].

Socioeconomic status (SES) was calculated based on occupation, income and parents' level of education [52,53]

Supplementary Information 2.

The following regression models were calculated:

(1) BDI-II=b0+b1*age+b2*sex+b3*BMI+b4* leptin level (lg);

(2) "anxious/depressed" scale of CBCL=b0+b1*age+b2*sex+b3*BMI+b4* leptin level (lg);

(3) "anxious/depressed" scale of YSR = b0+b1*age+b2*sex+b3*BMI+b4* leptin level (lg).

(4) BDI-II=b0+b1*age+b2*sex+b3*BMI+b4*leptin level (lg)+b5*(sex x leptin level (lg))+b6*(BMI x leptin level (lg));

(5) "anxious/depressed" scale of CBCL=b0+b1*age+b2*sex+b3*BMI + b4* leptin level (lg) + b5*(sex x leptin level (lg))+b6*(BMI x leptin level (lg));

(6) "anxious/depressed" scale of YSR=b0+b1*age+b2*sex+b3*BMI+b4* leptin level (lg) + b5*(sex x leptin level (lg)) + b6*(BMI x leptin level (lg));

(7) BDI-II=b0+b1*age+b2*sex+b3*BMI+b4 * leptin level (lg) + b5* leptin level (lg)^2;^

Analyses with leptin z-score as predictor instead of leptin level (lg):

(8) BDI-II=b0+b1*age+b2*sex+b3*BMI+b4* leptin z-score;

(9) "anxious/depressed" scale of CBCL=b0+b1*age+b2*sex+b3*BMI+b4* leptin z-score;

(10) "anxious/depressed" scale of YSR = b0+b1*age+b2*sex+b3*BMI+b4* leptin z-score.

**Supplementary Information 3.**

A sensitivity analysis of the effect of psychopharmacological medication on leptin levels (z-score) using UNIANOVA showed that patients without psychopharmacological medication had a mean leptin z-score of 1.294 (CI: 1.080; 0.508), while patients with psychopharmacological medication had a mean leptin z-score of 1.509 (CI: 1.101; 1.918). Both of these values were significantly higher than 0, but the pairwise group comparison was not significant (p = 0.358). The analysis on leptin (ln) showed, that there were no difference between groups with or without psychopharmacological medication (p=.227).

Supplementary Figure 1.


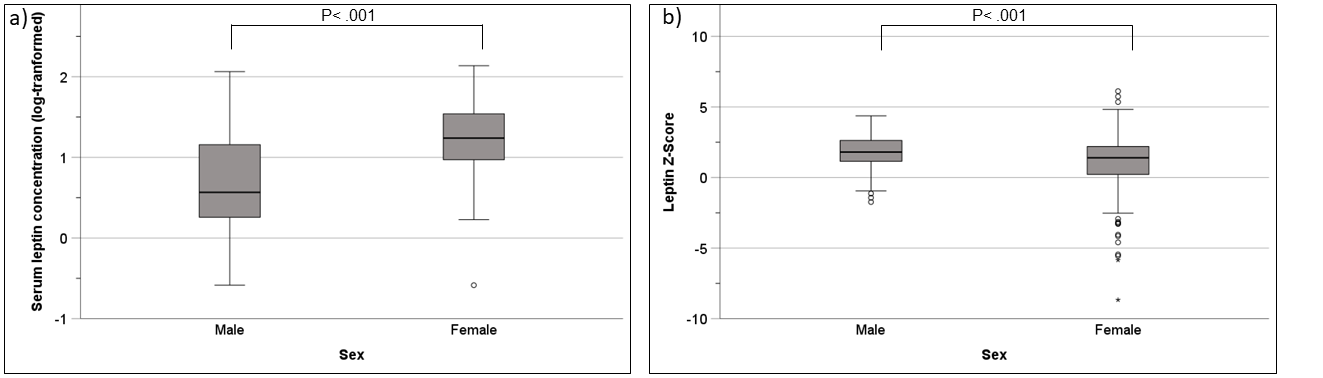


Sex differences between male (n=110) and female (n=228) adolescent patients with psychiatric disorders (excluding patients with AN) in a) leptin levels (ln), and b) leptin z-score. The mean measured leptin level in men was 11.1 (SD 18.22) and females 25.7 (SD 23.9). The mean leptin z-score in men was 1.78 (SD=1.16), median=1.81. Female patients had a mean leptin z-score of 1.12 (SD=1.97) and median=1.40.

Supplementary Table S1: Results of regression analysis with total BDI-II and the scale “anxious/depressed”of CBCL or YSR as dependent variable (n=363)

| **a) Results of regression analysis with total BDI-II as dependent variable (model 8)** | | | | | |
| --- | --- | --- | --- | --- | --- |
| Predictor | B [95%CI] | SE | Beta | t | Sig. ^a^ |
| Step 1 (*R^2^*=0.146) |  |  |  |  |  |
| Constant | -1.087 [-13.928-11754] | 6.530 |  | -0.166 | .868 |
| Sex | 9.196 [6.566-11.824] | 1.336 | 0.336 | 6.881 | **<.001** |
| Age | 1256 [0.489-2.023] | 0.390 | 0.157 | 3.221 | **.001** |
| BMI | 0.077 [-0.120-0.274] | 0.100 | 0.037 | 0.768 | .443 |
| Step 2 (*R^2^*=0.149) |  |  |  |  |  |
| Constant | -2.435 [-15.528-10.657] | 6.657 |  | -0.366 | .715 |
| Sex | 9.479 [6.797-12.162] | 1.364 | 0.347 | 6.950 | **<.001** |
| Age | 1.1259 [0.492-2.026] | 0.390 | 0.158 | 3.229 | **.001** |
| BMI | 0.107 [-0.098-0.321] | 0.104 | 0.052 | 1.024 | .306 |
| Leptin z-score | 0.345 [-0.310-0.999] | 0.333 | 0.054 | 1.036 | .301 |

| **b) Results of regression analysis with the t-score of the "anxious/depressed" scale of the CBCL as dependent variable (model 9).** | | | | | | |
| --- | --- | --- | --- | --- | --- | --- |
| Predictor | | B [95%CI] | SE | Beta | t | Sig. ^a^ |
| Step 1 (*R^2^*=0.036) | |  |  |  |  |  |
| Constant | 60.292 [49.967-70.616] | | 5.250 |  | 11.484 | **<.001** |
| Sex | 2.955 [0.842-5.068] | | 1.075 | 0.143 | 2.750 | .006 |
| Age | 0.336 [-0.281-0.953] | | 0.313 | 0.056 | 1.072 | .285 |
| BMI | 0.163 [0.004-0.332] | | 0.081 | 0.105 | 2.019 | .044 |
| Step 2 (*R^2^*=0.043) |  | |  |  |  |  |
| Constant | 58.623 [48.119-69.128] | | 5.342 |  | 10.975 | **<.001** |
| Sex | 3.306 [1.156-5.459] | | 1.094 | 0.160 | 3.021 | **.003** |
| Age | 0.340 [-0.275-0.955] | | 0.313 | 0.056 | 1.087 | .278 |
| BMI | 0.200 [0.035-0.365] | | 0.084 | 0.128 | 2.386 | .018 |
| Leptin z-score | 0.427 [-0.098-0.952] | | 0.267 | 0.088 | 1.598 | .111 |
| **c) Results of the regression analysis with the t-score of the "anxious/depressed" scale of the YSR as dependent variable (model 10).** | | | | | | |
| Predictor | | B [95%CI] | SE | Beta | t | Sig. ^a^ |
| Step 1 (*R^2^*=0.078) | |  |  |  |  |  |
| Constant | 48.202 [36.203-60.200] | | 6.101 |  | 7.901 | **<.001** |
| Sex | 5.480 [3.024-7.936] | | 1.249 | 0.223 | 4.388 | **<.001** |
| Age | 1.028 [0.312-1.745] | | 0.364 | 0.143 | 2.822 | .005 |
| BMI | 0.113 [-0.072-0.297] | | 0.094 | 0.061 | 1.202 | .230 |
| Step 2 (*R^2^*=0.086) |  | |  |  |  |  |
| Constant | 46.107 [33.906-58308] | | 6.204 |  | 7.432 | **<.001** |
| Sex | 5.920 [3.421-8.420] | | 1.271 | 0.241 | 4.658 | **<.001** |
| Age | 1.1033 [0.319-1.748] | | 0.363 | 0.144 | 2.843 | .005 |
| BMI | 0.159 [-0.032-0.350] | | 0.097 | 0.086 | 1.636 | .103 |
| Leptin z-score | 0.536 [-0.074-1.146] | | 0.310 | 0.093 | 1.727 | .085 |

*B* unstandardized regression coefficient; *CI* confidence interval; *SE* standard error from unstandardized regression coefficient; *Beta* standardized coefficient; *t* test statistic; *Sig*. significance; *R^2^* coefficient of determination; ^a^ p-values <0.005 are marked bold.
